# Mesenchymal Stem/Stromal Cells and Their Derivates in Acute Diseases: Emergency in the Post-COVID-19 Times

**DOI:** 10.3390/ijms22168395

**Published:** 2021-08-05

**Authors:** Francisco J. Vizoso, Silvia Fernández-Francos, Noemi Eiro

**Affiliations:** 1 Research Unit, Fundación Hospital de Jove, Av. Eduardo Castro, 161, 33290 Gijón, Spain; silviafernandezfrancos@gmail.com (S.F.-F.); noemi.eiro@hospitaldejove.com (N.E.); 2 Department of Surgery, Fundación Hospital de Jove, Av. Eduardo Castro, 161, 33290 Gijón, Spain; 3 Department of Anesthesiology, Fundación Hospital de Jove, Av. Eduardo Castro, 161, 33290 Gijón, Spain

**Keywords:** mesenchymal stem cells, extracellular vesicles, exosomes, acute diseases

The current coronavirus disease-19 (COVID-19) pandemic has strongly revived the pressing need to incorporate new therapeutic alternatives to deal with medical situations that result in a dramatic breakdown in the body’s normal homeostasis. However, independently of the pandemic, and despite the considerable progress in therapeutic pharmacology in recent times, approximately 10.4% of all emergency room visits end in hospitalization, with the highest rate of admissions to acute care units being due to the severity of the condition and the inability to treat it and discharge the patient [1]. In this context, the main causes of morbidity and mortality are acute respiratory failure caused by sepsis, acute heart failure, septic shock, acute kidney injury, and neurovascular disorders [2,3]. These processes display pathological mechanisms such as strongly proinflammatory responses, endothelial activation, microcirculatory dysfunction, ischemia reperfusion injuries, and oxidative stress [4], causing cells to die in vital injured organs. Classical pharmacological strategies often fail to address these pathological processes, whose mechanisms are very complex. Therefore, new perspectives must be pursued to more effectively handle the technological and scientific challenge of treating these catastrophic clinical situations.

Cell therapy has become one of the world’s largest emerging medical treatments. In addition, public interest in stem cell therapies continues to grow as regenerative science evolves, its clinical applications advance, and many cell therapy products are endorsed in the global market. Among these, mesenchymal stromal/stem cells (MSCs), discovered in the early 1970s, constitute a promising tool for disease management. According to the National Institutes of Health (http://www.clinicaltrial.gov/, accessed on 28 June 2021), there are currently over 1200 MSC clinical trials registered globally. The most common indications for MSC-based cell therapy include graft-versus-host disease (GvHD), osteoarthritis, systemic lupus erythematosus, liver cirrhosis, diabetes, Crohn’s disease, multiple sclerosis, and spinal cord injury [5]. However, MSC studies have also been reported for clinical applications in the acute setting by virtue of their beneficial anti-inflammatory, immunomodulatory, antifibrotic, proangiogenic, and regenerative effects [6,7]. In addition, since the escalation of COVID-19 outbreak into a pandemic status on 11 March 2020, numerous MSC-based studies have been documented combining COVID-19-related pneumonia and acute respiratory distress syndrome (ARDS) [8]. 

The utilization of MSC-based treatment for regenerative medical applications depends on their ability to promote immunomodulation and organ regeneration. Initially, the biological activity of MSC was ascribed to their ability to home within the injury site. However, it is known that only a small fraction of MSC may reach damaged tissues following systemic administration [9] because a majority of them are rapidly cleared by the spherocytosis phenomena. In addition, we must consider the limits of the low retention and survival rates of MSC in the context of acute lesions, such as myocardial infarction or acute kidney injury microenvironment. Nevertheless, it is now assumed that MSCs can act at the paracrine level by releasing bioactive factors, including cytokines, chemokines, growth factors, and microRNAs (miRNA). Moreover, in recent years, membrane-bound particles, known as extracellular vesicles (EV), have been recognized as an important MSC paracrine factor in addition to the abovementioned soluble factors. Solid experimental evidence shows that MSC EVs are able to mimic most of the biological actions of their parent cells [10]. EVs assist in intercellular signaling and maintain tissue homeostasis in the disease pathobiology. Researchers have characterized 9769 proteins, 2838 miRNAs, 3408 mRNAs (messenger RNA), and 1116 lipids in exosome cargo, the latter being one of the types in which EVs are classified [11]. 

Generally, MSC-secretome-derived products, such as conditioned medium or EVs, have the advantage of avoiding issues attributed to cell therapy, such as immune incompatibility, tumorigenicity, emboli formation, transmission of infections, and potential entry of MSC into the senescence. In contrast, secretome can be better evaluated for safety, dose, and potency, analogously to conventional therapeutic agents, and stored without having to apply presumably toxic cryopreservative agents. In addition, MSC-secretome-derived products are cheaper and more convenient for clinical use because the use of the secretome can save time and costs associated with expanding and maintaining clonal cell lines. Moreover, secretome for therapies can be prepared in large quantities ahead of time and be available for therapy if required [10]. On the other hand, MSC secretome, especially its EV, has shown beneficial effects due to their anti-inflammatory, antiapoptosis, antifibrosis, antioxidation, or angiogenesis promoting properties in several illness models, including acute diseases such as acute myocardial infarction (AMI) [12]. This progress can be significant in treating acute conditions of high clinical prevalence, such as AMI, sepsis, acute kidney injury, ARDS, and COVID-19.

With this array of untreated acute conditions, MSC can revolutionize patient care and management. MSC, along with the secretome and its derivatives, as well as the complex galaxy of intercellular signals can contribute to the optimal restoration of the abruptly truncated tissue homeostasis that characterizes these processes [13]. Therefore, a driving strategy aimed at the development of products derived from the secretome of MSC for their therapeutic application in acute diseases appears to be a major research stimulus worthy of economic investment. It is enough to recall the financing of other future prospects linked to regenerative medicine, which today, from a scientific insight, are no longer seen as so innovative. For example, there are about 800,000 cord blood units stored in state-owned banks, and 5,000,000 units held by private banks. In addition, by 2026, the global umbilical cord blood bank market is expected to achieve more than $20 billion, and the adult stem cell bank market will rise above $13 billion. In fact, several private companies have advertised services for the isolation and storage of different MSC biomaterials and reported on the potential future use of MSCs for a range of therapies, including acute diseases [14]. However, although phase I/II studies provide evidence for short-term safety, there has only been a limited number of clinical trials for various acute conditions demonstrating modest efficacy at present [15]. Therefore, future clinical trials will be required to cope with unanswered scientific questions, such as the optimal dose, route of administration, efficacy, effect durability, and adverse effects [16]. Dealing with these questions will require the execution of meticulously conducted, multicenter, placebo-controlled, randomized clinical trials with large sample sizes and longer patient follow-up in order to identify and elucidate clearly defined clinical outcomes. Furthermore, it is important to pursue new approaches and methods.

Even though the same molecular markers serve to identify and isolate MSC, they are highly heterogeneous depending on the donor and its origin in the human body, which corresponds to their role in the homeostasis of different tissue microenvironments [17]. Although this may be a drawback when standardizing their biological applications, it also offers the advantage of leveraging these different MSC conditions to optimize their therapeutic uses [18,19]. At the same time, while the safety of cell products is a priority and MSC studies are well documented, the lack of consistent and uniform methods that guarantee the safety and efficacy of the secretome and its derived products is a major concern. This could drastically slow the progression of this cell-free therapy into clinical use. Therefore, both basic and clinical studies should be encouraged in the near future in order to tackle not only future pandemics with greater optimism but also current conundrums that categorically demand new strategies, such as therapeutic alternatives for acute diseases based on MSC.

## Data Availability

Not applicable.
